# First-line treatment for lung cancer among Japanese older patients: A real-world analysis of hospital-based cancer registry data

**DOI:** 10.1371/journal.pone.0257489

**Published:** 2021-09-20

**Authors:** Shoko Noda-Narita, Asuka Kawachi, Ayako Okuyama, Ryo Sadachi, Akihiro Hirakawa, Yasushi Goto, Yasuhiro Fujiwara, Takahiro Higashi, Kan Yonemori

**Affiliations:** 1 Department of Breast and Medical Oncology, National Cancer Center Hospital, Tokyo, Japan; 2 Center for Cancer Registries, Center for Cancer Control and Information Services, National Cancer Center, Tokyo, Japan; 3 Department of Biostatistics and Bioinformatics, Graduate School of Medicine, The University of Tokyo, Tokyo, Japan; 4 Department of Thoracic Oncology, National Cancer Center Hospital, Tokyo, Japan; Chang Gung Memorial Hospital and Chang Gung University, Taoyuan, TAIWAN

## Abstract

Aging of the population has led to an increase in the prevalence of cancer among older adults. In Japan, single agent chemotherapy was recommended for advanced non-small cell lung cancer (NSCLC) for those, who were aged ≥75 years, while the Western guidelines did not recommend a specific regimen. In clinical practice, physicians are required to decide the treatment based on a lack of enough evidence. This study aimed to examine the prescribing patterns of first-line chemotherapy according to age in the real-world practice. Data from the survey database of Diagnostic Procedure Combination and hospital-based cancer registries of designated cancer centers nationwide were used. The first-line chemotherapy regimens among 9,737 patients who were diagnosed with advanced lung cancer between January and December 2013, were identified and compared based on age. We found that the proportion of patients receiving chemotherapy decreased with age; 80.0%, 70.4%, 50.6%, and 30.2% of patients aged 70–74, 75–79, 80–84, and ≥ 85 years, respectively, received chemotherapy. Among them, platinum doublets were prescribed for 62.7% of the patients who were aged ≥ 70 years, and 60.7% of the patients who were aged ≥ 75 years with no driver mutations in NSCLC; only 37.6% of them received single agents. Patients who were aged ≥ 80 years also preferred platinum doublets (35.6%). Carboplatin was commonly prescribed in all age groups; only 28.4% of those receiving platinum doublets selected cisplatin. In this study, platinum doublets were identified as the most commonly prescribed regimen in those who were aged ≥ 70 years. Despite recommendations of Japanese guidelines for NSCLC, 60.7% of those who were aged ≥75 years received platinum doublets. Additionally, patients who were aged ≥ 80 years also received systemic chemotherapy, including platinum doublets; age did not solely influence regimen selection.

## Introduction

Lung cancer is one of the most common cancers affecting the older population in Japan; the median age at diagnosis is 70 years, and one-third of the patients are over 80 years old [1]. In view of the relatively poorer outcomes and risks of severe toxicity, treatment options for older patients with advanced lung cancer remain a subject of discussion. A single phase 3 clinical trial has suggested that carboplatin (CBDCA) containing doublet therapy prolongs overall survival compared to that with monotherapy in older patients [2]; however, this finding has not been validated by those from other studies. Current guidelines from the Western population indicate that the selection of chemotherapy should not be based on age alone, as none of the previous prospective clinical trials have solely identified age as a risk factor for poorer outcomes [3–6]. However, older patients are under-represented in clinical trials, and those who are recruited have fewer comorbidities, and do not always represent the real-world heterogeneous populations.

The European Society of Medical Oncology (ESMO) guideline for non-small cell lung cancer (NSCLC) recommends the use of different regimens in older patients [5]. It recommends the use of CBDCA based platinum doublets for patients who are aged ≥ 70 years, based on evidence from a systematic review of thirteen clinical trials [7]; however, the feasibility of this treatment among patients who are aged ≥ 80 or 90 years has not been verified. Other Western guidelines, including those by the American Society of Clinical Oncology (ASCO), do not recommend specific regimens for older patients.

Japan is at the forefront of an aging society and has actively optimized medical care for older adults. The guidelines of the Japan Lung Cancer Society categorize patients who are aged ≥75 years as older patients and recommend the use of specific regimens other than those prescribed for younger patients [8]. The guidelines recommend the use of third generation single agent chemotherapy, such as docetaxel (DTX), gemcitabine (GEM), and vinorelbine (VNR), in older patients with NSCLC with no driver mutations. These recommendations have been based on evidence from specific clinical trials among older adults, which demonstrated superior outcomes with VNR compared to best supportive care (BSC), and better outcomes with GEM and DTX compared to VNR [9–11]. The guidelines also recommend tyrosine kinase inhibitors (TKIs) for older and younger patients with specific biomarkers. Among those with small cell lung cancer (SCLC), the guidelines continue to recommend platinum doublets for older adults; however, the feasibility of their administration in oldest old patients remains unclear.

This study focused on this gap between the Western and Japanese guidelines, and aimed to investigate the selection of regimens for advanced lung cancer patients based on age and histology, using two large real-world nationwide databases from Japan. The data of the Diagnostic Procedure Combination (DPC) survey, that encompasses all services, were combined with those of hospital-based cancer registries, which collect basic information on all patients with cancer encountered in hospitals. Although the data do not fully cover the relevant clinical factors or outcomes such as performance status (PS), complications, and survival, it is the most extensive data covering 70 percent of the newly diagnosed patients with cancer annually and representative to illustrate the demography of treatment choice in Japan. We focused on the real-world choice of the detailed regimen, especially of cytotoxic agents, according to age. We believe that this study will be of particular value to those routinely involved in the selection of treatment for older adults.

## Materials and methods

### Data resources

**Fig 1:**
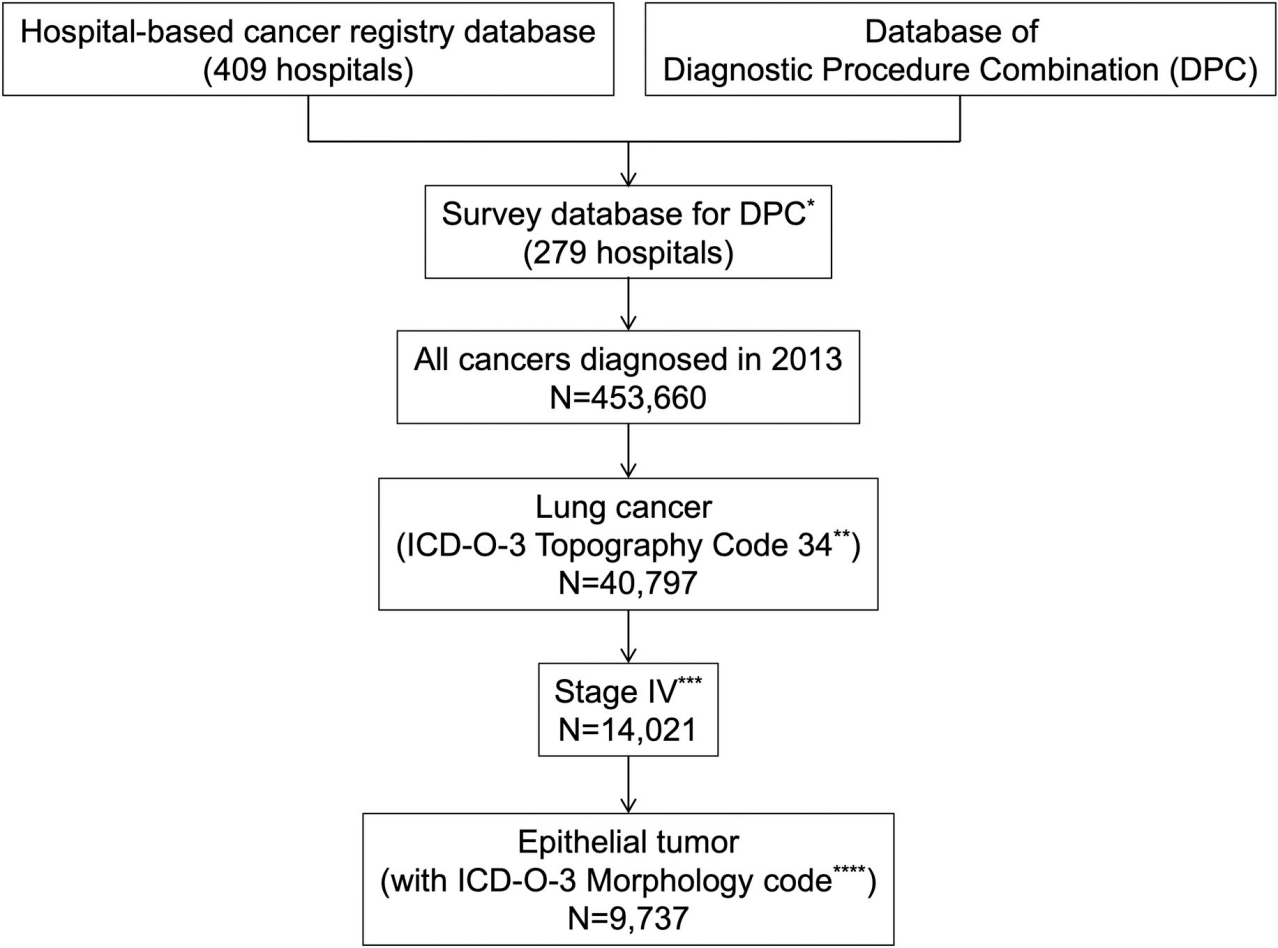
Flow chart for selection of the study population. The survey database for Diagnostic Procedure Combination (DPC) was combined with the hospital-based cancer registry and the DPC. * Patients who had a diagnosis and a selected first choice of treatment including best supportive care, were included in the survey database. ** ICD-O-3 topography codes C34.0 (main bronchus), C34.1 (upper lobe of lung), C34.2 (middle lobe of lung), C34.3 (lower lobe of lung), C34.8 (overlapping lesion of lung), C34.9 (lung, not otherwise specified) were identified. *** Patients with de novo stage IV disease as per the UICC classification were identified. **** Adenocarcinomas (ICD-O-3 morphology codes 8140, 8250, 8551, 8260, 8265, 8230, 8253, 8254, 8480, 8333, and 8144), squamous cell carcinomas (8070, 8071, 8072, and 8083), neuroendocrine carcinomas (8041, 8045, and 8013), carcinoid tumors (8240, 8249, 8012, and 8560), sarcomatoid carcinomas (8022, 8032, 8031, 8980, and 8972), other and unclassified carcinomas (8082 and 8023), and salivary gland-type tumors (8430, 8200, and 8562) were identified.

We obtained data on the study participants from two independent nationwide databases, namely, the hospital-based cancer registries of the nationwide designated cancer care hospitals (DCCHs) and the DPC. Details regarding both databases have been mentioned elsewhere [12, 13]. In summary, the Japanese government mandated hospital-based cancer registries for DCCHs in 2007 to ensure effective cancer control and monitor their current status of cancer care. DCCHs are hospitals that support cancer care and provide evidence-based standardized care to patients in a particular area. In 2013, 397 DCCHs were registered to provide care for up to 70% of all new patients with cancer in Japan [12]. Cases were coded based on the International Classification of Diseases for Oncology, 3rd edition (ICD-O-3). The DPC is a case-mix classification system for determining the daily amount of reimbursement from the national insurance to hospital inpatient services [13]. Hospitals opting for reimbursement as per the DPC schedule are required to participate in a related survey, which collects all information on health services provided in both, in-hospital and outpatient settings. These health services are coded as per the fee-for-service health insurance codes; these include all pharmaceutical products covered in Japan. We used these pharmaceutical data to analyze the provision of chemotherapy and regimens. The combined database used in this study was obtained from 279 (68.2%) DCCHs (Fig 1) [14].

The database used in this study was combined data collected of every cancer type for the different purposes of monitoring the current status of cancer care and reimbursement of hospital impatient services. The collected clinical factors in the database were limited in this reason, and the factors associating with treatment decisions such as predictive biomarkers, PS, and comorbidities were not included.

### Identification of lung cancer

We obtained the data of all patients who were newly diagnosed with advanced lung cancer (de novo stage IV) in 2013, from the national database of hospital-based cancer registries. We defined lung cancer based on topographic code C34 of the ICD-O-3. Histology was defined based on the morphology codes; the codes for adenocarcinoma were 8140, 8250, 8551, 8260, 8265, 8230, 8253, 8254, 8480, 8333 and 8144. Those for squamous cell carcinoma were 8070, 8071, 8072, and 8083, and that of adenosquamous cell carcinoma was 8560; the codes for small cell carcinoma were 8041 and 8045, while those for neuroendocrine and large cell carcinomas were 8013 and 8012, respectively. The codes for carcinoid tumors were 8240 and 8249, and those for sarcomatoid carcinoma were 8022, 8032, 8031, 8980, and 8972; and the codes for other/unclassified carcinomas were 8082, 8023, 8430, 8200, and 8562.

For this analysis, we defined NSCLC as adenocarcinoma, squamous cell carcinoma, adenosquamous cell carcinoma, and sarcomatoid carcinoma, and SCLC as small cell, neuroendocrine, and large cell carcinomas.

### Definition of treatment

This analysis focused on first-line chemotherapy. We obtained information regarding all anti-tumor agents approved for lung cancer in Japan. However, the precise doses of those agents were not available from the data. The agents first received after diagnosis were defined as first-line treatment. We defined regimens containing cisplatin (CDDP), CBDCA, or nedaplatin in combination with other agents as platinum doublets. The cytotoxic agents suggested for metastatic or unresectable lung cancer as per the guidelines of the Japan Lung Cancer Society were defined as single-agent therapies. TKIs included gefitinib, erlotinib, and crizotinib. Not-recommended chemotherapy included all the anti-tumor agents, that were approved for lung cancer in Japan, despite not being recommended in the guidelines. BSC included all treatment except chemotherapy, such as palliative surgery and palliative radiation therapy.

### Statistical analysis

No statistical tests were conducted in this study. Descriptive analyses were performed for all the data collected from the two databases. All statistical analyses were performed using the SAS version 9.4 (SAS Institute, Inc., Cary, NC, USA) software package.

### Ethical considerations

This research was approved by the NCCH Institutional Review Board (2017–402). All the data used were provided by the Review Committee at the Center for Cancer Control and Cancer Information Service at the National Cancer Center. The need for informed consent was waived due to the retrospective nature of the study design.

## Results

### Patient characteristics

The data of 9,737 patients who were diagnosed with advanced lung cancer in Japan between January and December 2013, were analyzed in this study. The median age of the cohort was 70 years, and 71.9% were male; 60.2%, 17.6%, and 18.7% of patients had pathological diagnoses of adenocarcinoma, squamous cell carcinoma, and small cell carcinoma, respectively (Table 1). The median age of patients with each histology was 70, 72, and 71 years, respectively, and the proportion of male patients with adenocarcinoma, squamous cell carcinoma, and small cell carcinoma was 63.4, 85.9, and 84.6%, respectively.

**Table 1:** Patient characteristics of each age group.

	All n (%)	< 50	50–55	55–60	60–65	65–70	70–75	75–80	80–85	≥ 85
**All**	9737	365	342	609	1390	1883	1880	1626	1112	530
**Sex**										
**Male**	7005 (71.9)	221	242	438	1008	1392	1402	1183	783	336
**Female**	2732 (28.1)	144	100	171	382	491	478	443	329	194
**Histology**										
**Adeno**	5865 (60.2)	278	238	399	892	1118	1050	906	636	348
**Squamous**	1714 (17.6)	30	40	81	201	335	373	322	239	93
**Small**	1816 (18.7)	38	49	104	234	377	389	345	205	75
Others*	342 (3.5)	19	15	25	63	53	68	53	32	14

Adeno, adenocarcinoma; Squamous, squamous carcinoma; Small, small cell carcinoma.

*Other histologies included adenosquamous carcinoma (0.3%), neuroendocrine carcinoma (1.1%), large cell carcinoma (1.1%), carcinoid tumor (0.1%), and pleomorphic/spindle carcinoma (0.8%).

### Treatment choices by age

**Fig 2:**
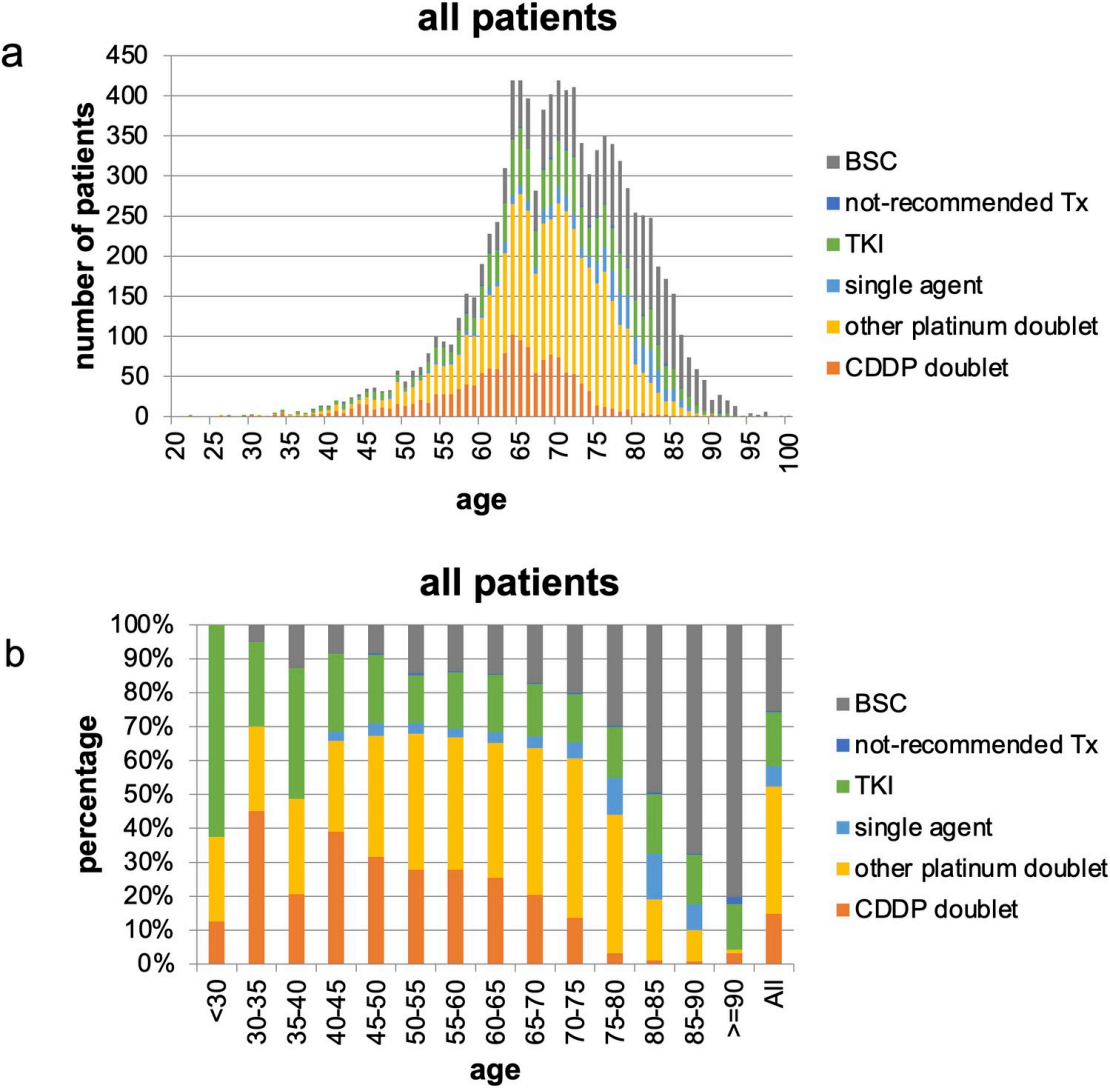
First-line treatment choice in all patients. (a) number of patients and (b) percentage of patients. CDDP, cisplatin; TKI, tyrosine kinase inhibitor; not-recommended Tx, not-recommended chemotherapy; BSC, best supportive care.

Overall, 74.7% of the patients received systemic chemotherapy, and the remainder received BSC. The proportion of patients who received systemic chemotherapy in the 70, 75, and ≥ 80-year age groups was 65.5%, 57.1%, and 44.0%, respectively (Fig 2).

Platinum doublets were selected for 62.7% of the older patients who were aged ≥70 years and received chemotherapy; this was almost similar to the proportion in the total population (76.3%). CDDP was only selected for 9.6% of the patients who were aged ≥ 70 years; the frequency lowered with age (3.6% and 2.4% in aged ≥ 75 and 80-year groups, respectively). CBDCA was often preferred even in younger patients than CDDP and was selected for 84.7% of the patients who were aged ≥ 70 years (Fig 2).

The treatment choices gradually changed with increasing age, and older patients with SCLCs received chemotherapy more frequently than those with NSCLCs. Among those with NSCLCs and SCLCs, the majority in the early and late 80s, respectively, received BSC (S1 Fig). Among the patients who received cytotoxic chemotherapy as the first-line treatment for NSCLC, only 37.6% of those who were aged ≥ 75 years received single agents, as recommended by the Japanese guidelines.

### Treatment choice by histology

**Fig 3:**
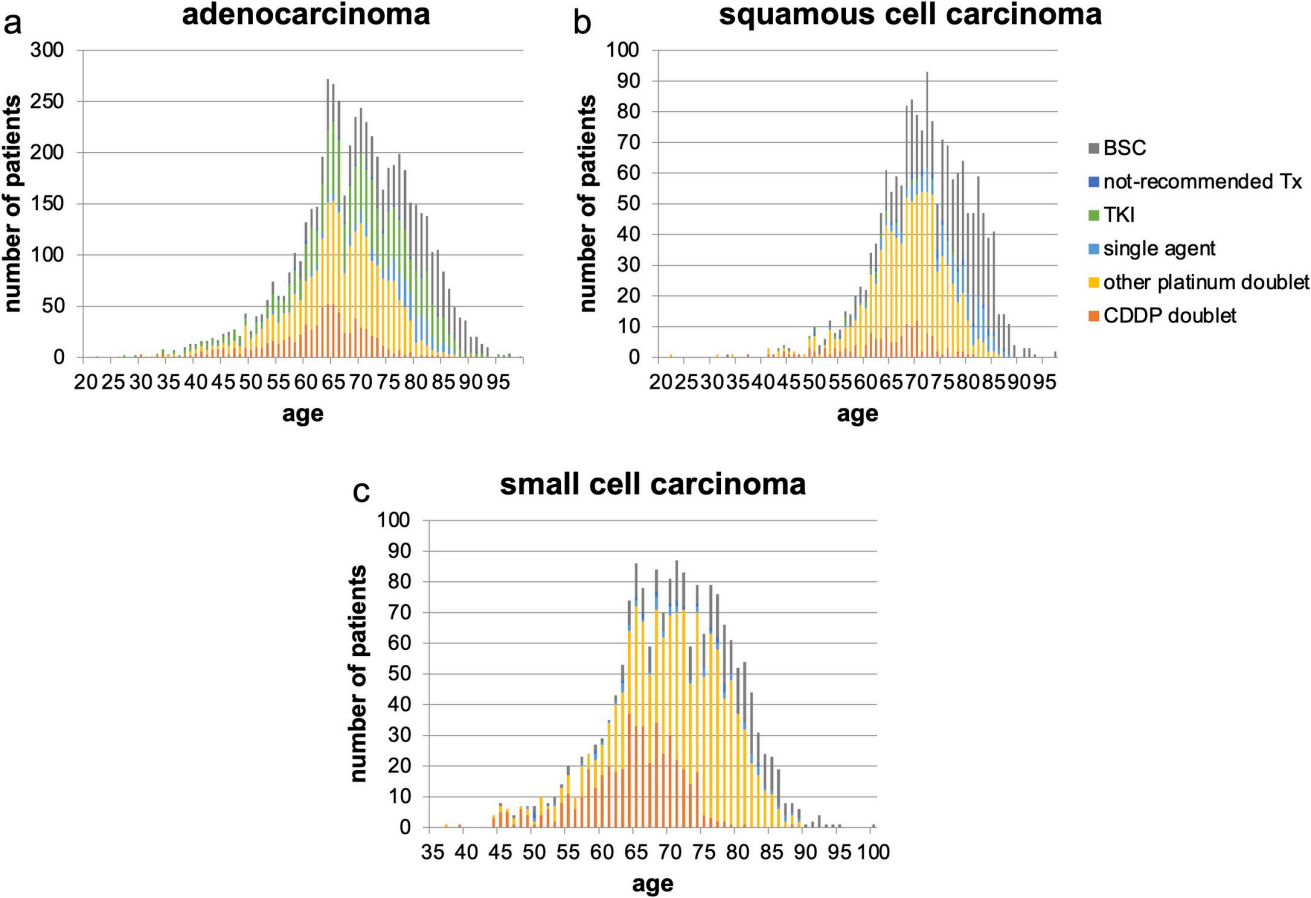
First-line treatment choice by histology. (a) adenocarcinoma, (b) squamous cell carcinoma, and (c) small cell carcinoma. CDDP, cisplatin; TKI, tyrosine kinase inhibitor; not-recommended Tx, not-recommended chemotherapy; BSC, best supportive care.

**Fig 4:**
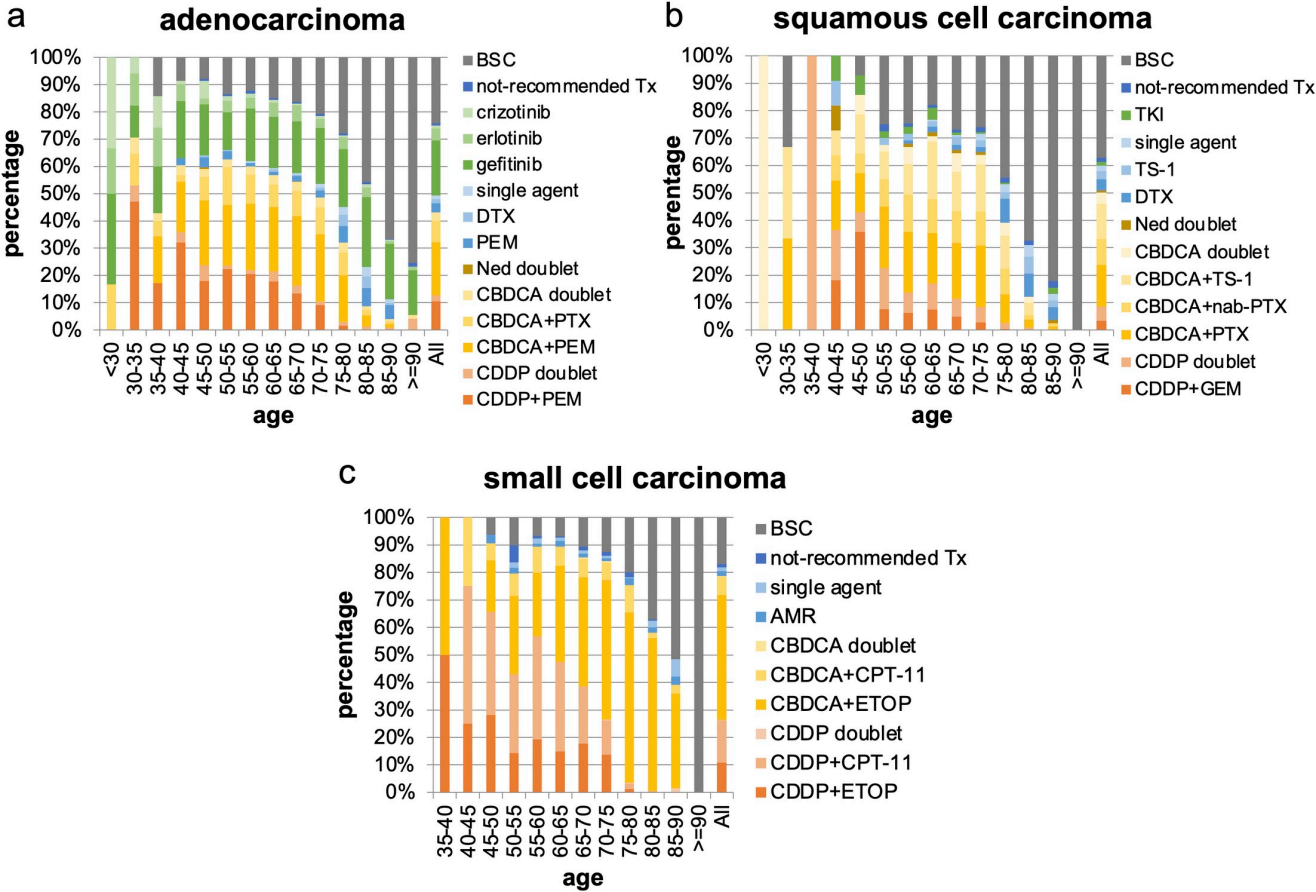
First-line treatment regimens by histology. (a) adenocarcinoma, (b) squamous cell carcinoma, and (c) small cell carcinoma. CDDP, cisplatin; PEM, pemetrexed; CBDCA, carboplatin; PTX, paclitaxel; Ned, nedaplatin; DTX, docetaxel; not-recommended Tx, not-recommended chemotherapy; BSC, best supportive care; GEM, gemcitabine; nab-PTX, nab-paclitaxel; ETOP, etoposide; CPT-11, irinotecan; AMR, amrubicin.

**Fig 5:**
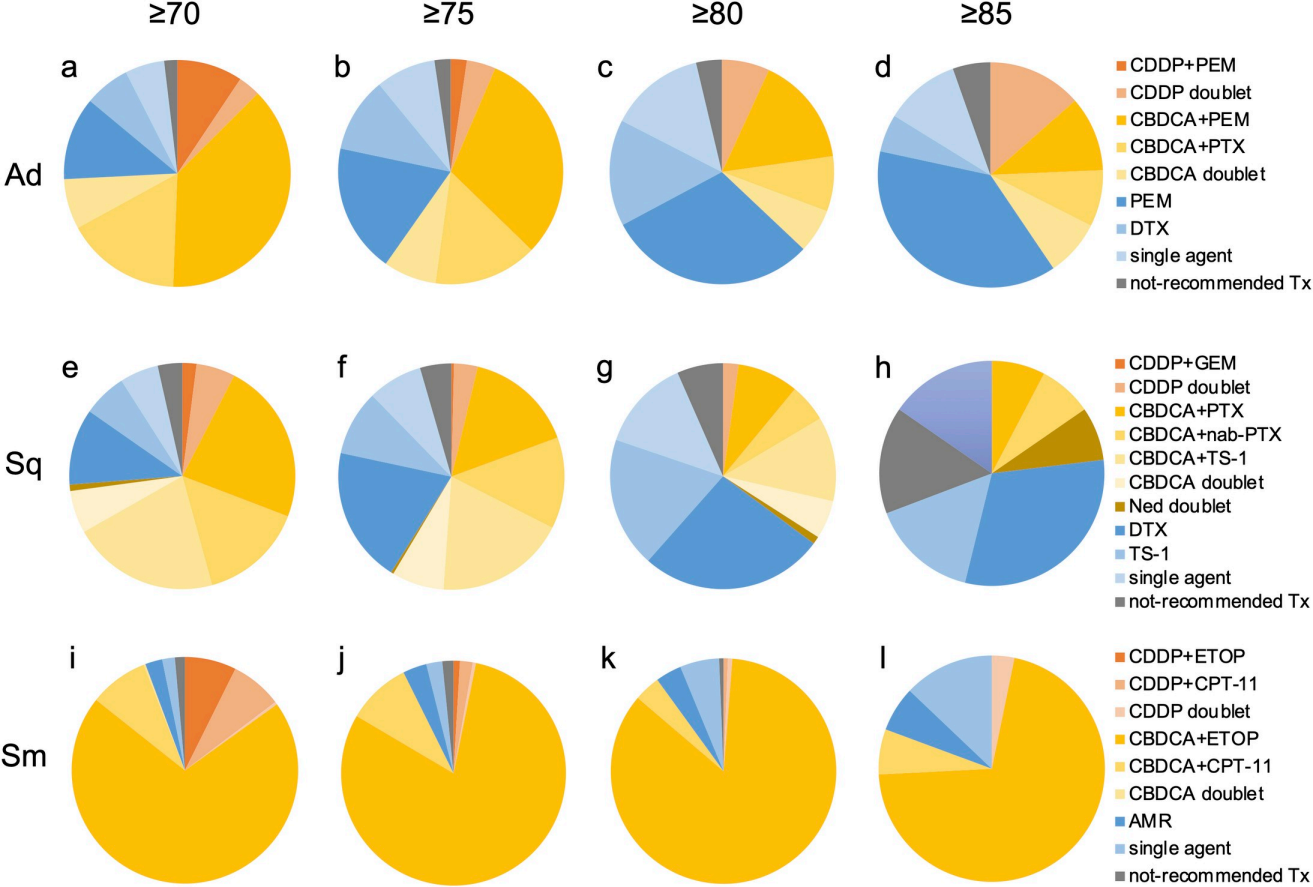
First-line choice of cytotoxic chemotherapy in older patients. (a)-(d) adenocarcinoma, (e)-(h) squamous cell carcinoma, and (i)-(l) small cell carcinoma. Ad, adenocarcinoma; Sq, squamous cell carcinoma; Sm, small cell carcinoma; CDDP, cisplatin; PEM, pemetrexed; CBDCA, carboplatin; PTX, paclitaxel; DTX, docetaxel; not-recommended Tx, not-recommended chemotherapy; GEM, gemcitabine; nab-PTX, nab-paclitaxel; Ned, nedaplatin; ETOP, etoposide; CPT-11, irinotecan; AMR, amrubicin.

A total of 5,865 patients were diagnosed with adenocarcinoma, and 75.8% of them received systemic chemotherapy. Among those who received chemotherapy, 33.3% received driver mutation-based targeted therapy, and gefitinib was the most commonly (78.5%) prescribed TKI. The proportion of patients receiving TKIs did not differ by age. Of those patients who received TKIs, 95.4% received epidermal growth factor receptor (EGFR) inhibitors, and 4.6% received anaplastic lymphoma kinase inhibitors. The majority of the patients receiving EGFR-TKIs chose gefitinib. This trend was observed among both older patients (86.5%) and young patients who were aged < 75 years (74.2%). Further, among those who received chemotherapy, 57.0% received platinum doublets, and the most common agent combined with platinum was pemetrexed (PEM) (69.7%) (Figs 3A, 4A and 5A–5D). Notably, DTX, which is recommended as the first choice of treatment for patients who are aged ≥ 75 year by guidelines, was not always selected, and PEM was more frequently prescribed than DTX.

A total of 1,714 patients were pathologically diagnosed with squamous cell carcinoma and 62.8% received systemic chemotherapy. Among them, 79.7% received platinum doublets, and the agents most commonly combined with platinum were paclitaxel (PTX), TS-1, and nab-paclitaxel (nab-PTX) (30.7%, 25.8%, and 18.9%, respectively). The use of CDDP was lower than in the other histologies; among those receiving platinum doublets, only 16.8% received CDDP (Figs 3B, 4B, 5E–5H and S2).

A total of 1,816 patients were pathologically diagnosed with small cell carcinoma and 83.0% received systemic chemotherapy. Among them, 94.8% received platinum doublets; the agents most commonly combined with platinum were etoposide (ETOP) and irinotecan (CPT-11) (71.4% and 28.1%, respectively). Platinum doublets were more commonly selected for patients who were pathologically diagnosed with small cell carcinoma than for those with other histologies. The trend was also observed in the oldest old patients who were aged ≥ 80 years (Figs 3C, 4C, 5I–5L and S2).

## Discussion

The current study intended to identify treatment choices for older patients with lung cancer in the Japanese population. The findings suggest that platinum doublet, which is the standard chemotherapy for younger patients, is also preferred for older patients; platinum doublets were selected in 62.7% of patients receiving chemotherapy at ages ≥70 years and in certain patients who were even aged ≥ 80 years. The majority of patients undergoing platinum doublet therapy received CBDCA. This trend was observed among both older patients (84.7%) and young patients who were aged < 70 years (62.3%). The proportion of platinum doublet therapy also varied based on histology; patients with SCLC selected platinum doublet therapy more frequently than those with NSCLC (77.7% vs. 45.5%).

This study was the first to elucidate the chemotherapy regimens used for metastatic lung cancer in Japan, using a large-scale database comprising a nationwide registry and health service data. We focused on the age and evaluated the patterns of chemotherapy regimens used in each generation. In Japan, only a few studies have reported on the treatment patterns in older adults with lung cancer [15, 16]; the chemotherapy regimens selected in the real-world scenario have remained unclear. Wang et al. compared the patterns of chemotherapy regimen selection between patients who were aged ≥ 75 years and those who were younger, with particular focus on the use of platinum agents in NSCLC [15]. However, the relation between the chemotherapy regimens and the age per year was not reported, and data for SCLC were lacking.

In the other countries, despite several real-world analyses on lung cancer, few studies have explored the chemotherapy regimens prescribed in the older population. Lee YG et al. compared the survival benefit of platinum doublet and single agent chemotherapy in older patients with NSCLC [17]. They presented the data of nationwide population-based outcomes in Korea; 7,298 older patients who were aged ≥ 70 years and received cytotoxic chemotherapy for advanced NSCLC were included. The findings suggested that platinum doublets conferred survival benefits over single agent chemotherapy (median overall survival: 10.8 vs. 9.8 months) and that they were also effective in the older population. However, the study excluded patients who received BSC; the efficacy of intensive chemotherapy may have merely reflected the baseline characteristics. Another study based on Surveillance, Epidemiology, and End Result (SEER) registry data, included 143,548 patients with NSCLC; it reported on the survival benefit of individual agents including platinum, DTX, PEM, and bevacizumab; however, the usage patterns in the older population were not evaluated [18]. In SCLC, only one study based on SEER registry data evaluated the chemotherapy regimens administered in patients who were aged > 65 years; however, it focused on the cost-effectiveness of palliative chemotherapy in the older population, and did not provide data on the tendency in individual age-groups [19].

Notably, this study demonstrated the preference of CBDCA containing platinum doublets over single agent chemotherapy even in the older population; the Japanese guidelines recommend single agent chemotherapy in this population. In 2019, the JCOG 1210 trial, which was conducted in Japan, demonstrated the non-inferiority of combined CBDCA and PEM to DTX in patients with non-squamous NSCLC who were aged ≥ 75 years [20]. This is reflected by the Japanese guidelines after 2019; however, CBDCA containing regimens were already preferred in real-world clinical practice in 2013. Furthermore, the standard treatment of NSCLC currently includes immune-checkpoint inhibitors (ICIs) with platinum doublets. Subset analysis of KEYNOTE-189 demonstrated inferior efficacy of triplet regimen of CBDCA, PEM, and pembrolizumab in patients who were aged ≥ 75 years. The JCOG 1210 and our study demonstrated consensus between trial and real-world settings, in that CBDCA and PEM were found to be the standard regimen even for the older population. The efficacy or real-world use of additional ICIs is also an issue of further interest.

Our study also demonstrated that the patients and physicians selected the treatments not only by the guidelines but according to the toxicity and accessibility of the treatment. In this study, we found out the preference of gefitinib among EGFR-TKIs, although gefitinib and erlotinib are both recommended in the Japanese guidelines in 2013. The patients might prefer the toxic profile of gefitinib with fewer subjective symptoms, such as skin rash and diarrhea, which are feasible even for PS 3–4 patients [21]. We also found out the preference of ETOP over CPT-11 as the agent combined with platinum for the patients diagnosed with small cell carcinoma, although the Japanese guidelines recommend CPT-11 based on the Japanese clinical trial [22]. As demonstrated in this study, the patients and physicians preferred CBDCA over CDDP even in the young patients, and therefore ETOP might be selected as the agent recommended to use in combination with CBDCA. The toxicity profile of ETOP with fewer subjective symptoms, such as diarrhea and nausea, might be preferred as well. In addition, the administration schedule of ETOP of consecutive 3 days might be suitable for hospital treatment.

Our study has several limitations. The first is that our database was limited to hospitals that voluntarily submitted DPC data to National Cancer Center. Although the hospital-based cancer registry is mandatory for DCCHs, the submission of the DPC was not mandatory. Nonetheless, our data included at least 270 facilities, and 9,737 patients with stage IV lung cancer. To the best of our knowledge, this is one of the largest reports on patterns of care in this setting. Thus, our study analyzed the best real-world data available in Japan. Second, we used the data of patients, who were diagnosed before 2018, which was prior to the approval of ICIs as first-line treatment for advanced lung cancer. This is because the dataset collected after 2018 is not still accessible for research purpose. We agree that the dataset is old, and the prognosis of lung cancer has remarkably improved with ICIs. However, it is also true that the most patients with advanced lung cancer need to undergo cytotoxic chemotherapy at some point. Even after the emergence of immunotherapy, it is necessary to be equipped with the knowledge of the optimal agent for cytotoxic chemotherapy for each patient, especially older patients, since the agents in cytotoxic chemotherapy have respective toxicity. Third, this survey did not fully cover the data associated with pertinent clinical factors during analysis, including PS and complications; in addition, we could not report on the dose reduction and outcomes of each chemotherapy regimen. However, these data are the most extensive data in Japan, covering 70 percent of the newly diagnosed cancer patients. Further investigations are necessary to assess the survival impact associated with of each clinical factor.

## Conclusion

This study included data from hospital-based cancer registries of the nation-wide DCCH and DPC; these are the largest real-world databases for evaluating treatment choices in routine clinical practice in Japan. The study illustrated the treatment choices in older patients and showed that platinum doublet therapy was prescribed for most patients who were aged ≥ 70 years. Even the patients with NSCLC who were aged ≥ 75 years frequently chose platinum doublets. The findings do not concur with the established guidelines in Japan. We also found that the regimen choice varied according to the individuals, and physicians did not select treatment solely based on age. Further, we also found that the real-world use of CBDCA regimens in older patients preceded the evidence of clinical trials. These real-world data provide valuable insight into the use of systemic chemotherapy in older patients with lung cancer.

Systemic chemotherapy for lung cancer has currently taken a major step forward with the addition of ICIs; the possibility of treatment optimization, and the various options available for newly-detected oncogenic driver mutations, are expected to change treatment choices in the older population in the next decade. Further analysis of real-world data is necessary to improve decision-making for treating older individuals with lung cancer.

## Supporting information

S1 Fig First-line treatment choices for each lung cancer type. (a, b) number of patients and (c, d) percentage of patients. NSCLC, non-small cell lung cancer; SCLC, small cell lung cancer; CDDP, cisplatin; TKI, tyrosine kinase inhibitor; not-recommended Tx, not-recommended chemotherapy; BSC, best supportive care.
(TIF)

S2 Fig First-line treatment choices of cytotoxic chemotherapy by histology. (a) adenocarcinoma, (b) squamous cell carcinoma, and (c) small cell carcinoma. CDDP, cisplatin; CBDCA, carboplatin.
(TIF)

S1 Dataset (XLSX)
