# Supplementary figures and images for: First-line treatment for lung cancer among Japanese older patients: A real-world analysis of hospital-based cancer registry data
Source: PLoS One. 2021 Sep 20;16(9):e0257489. doi: 10.1371/journal.pone.0257489 (PMC8452055; doi:10.1371/journal.pone.0257489)

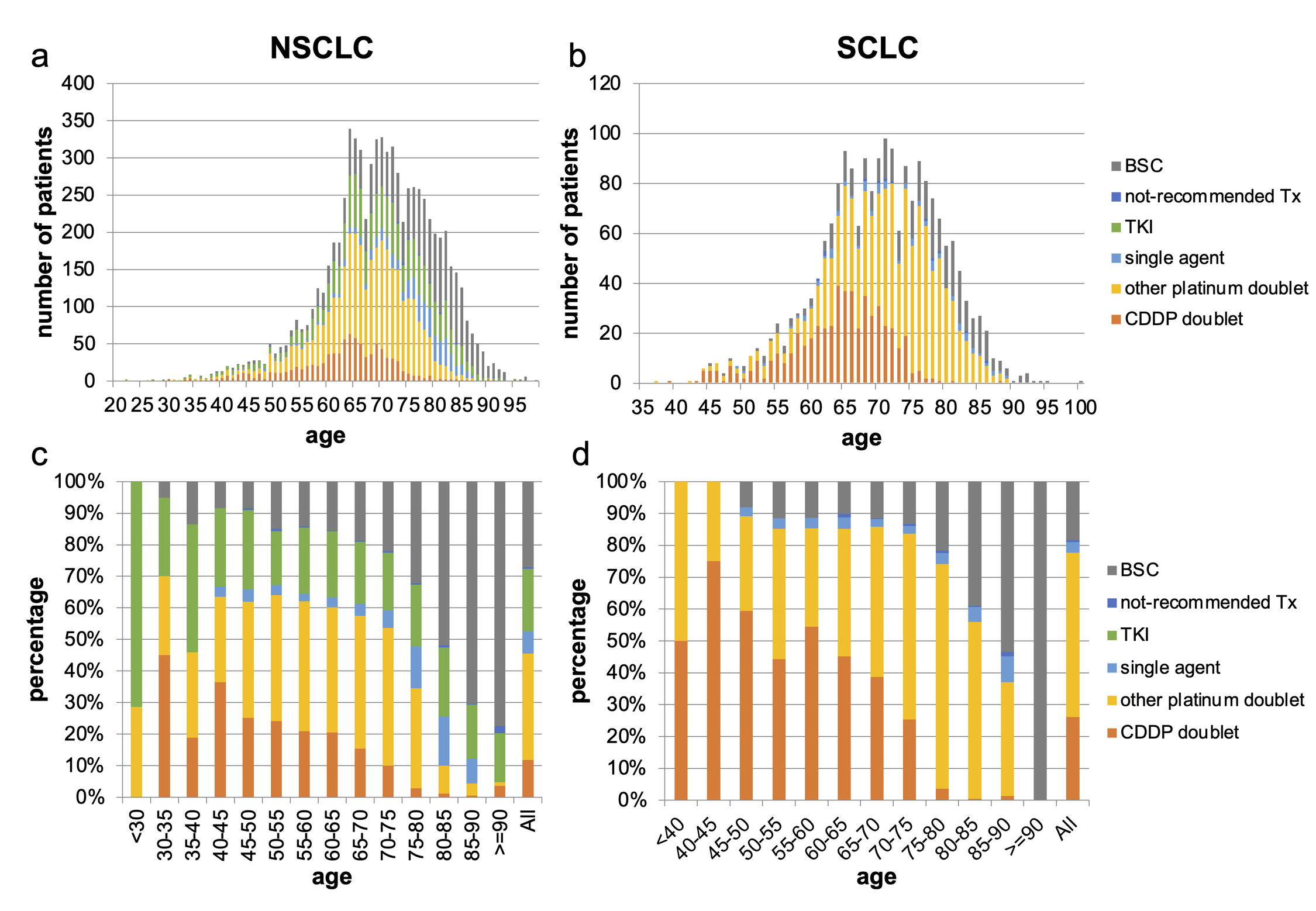

Supplement: S1 Fig — (a, b) number of patients and (c, d) percentage of patients. NSCLC, non-small cell lung cancer; SCLC, small cell lung cancer; CDDP, cisplatin; TKI, tyrosine kinase inhibitor; not-recommended Tx, not-recommended chemotherapy; BSC, best supportive care. (TIF) [file pone.0257489.s001.tif]

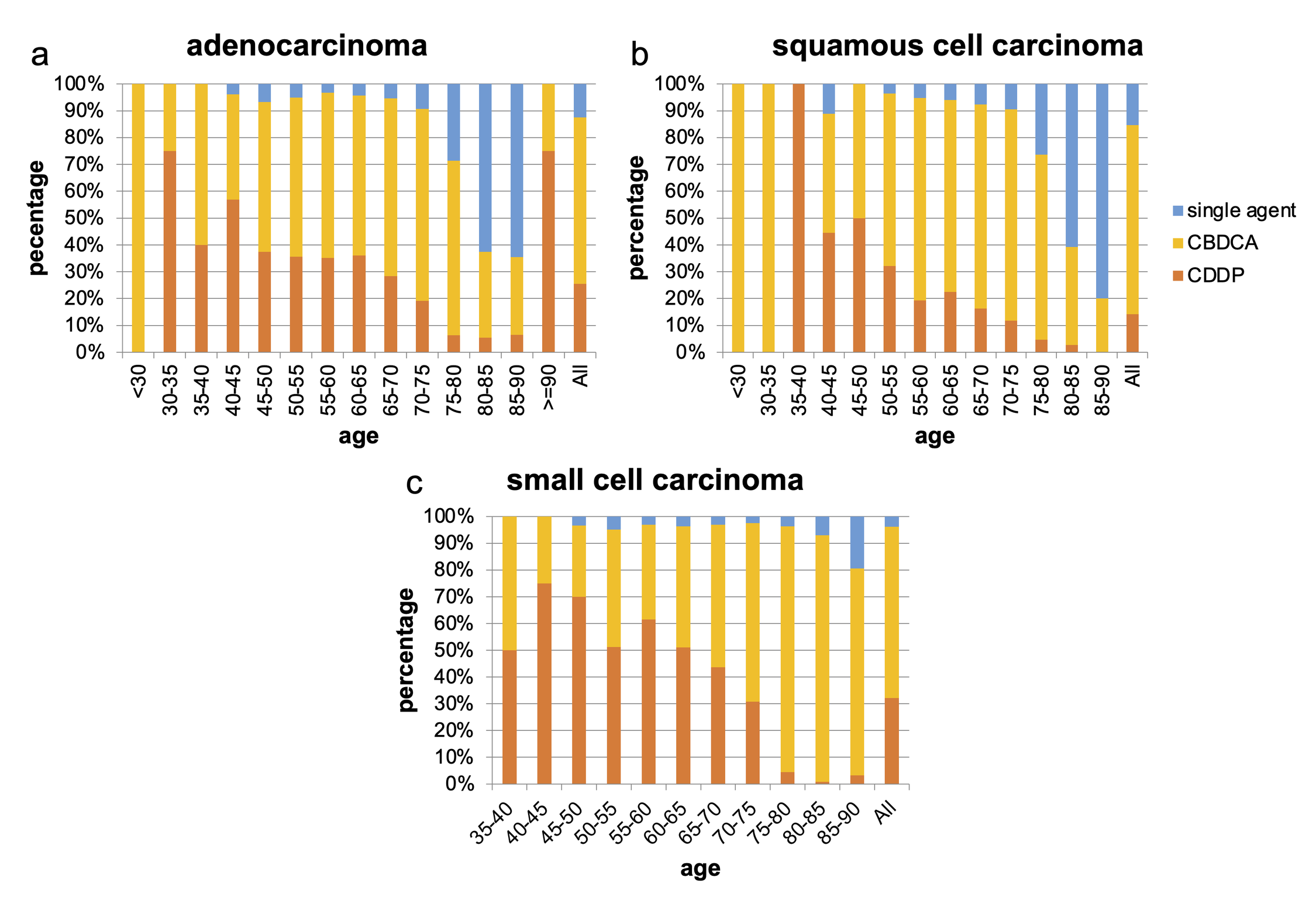

Supplement: S2 Fig — (a) adenocarcinoma, (b) squamous cell carcinoma, and (c) small cell carcinoma. CDDP, cisplatin; CBDCA, carboplatin. (TIF) [file pone.0257489.s002.tif]
